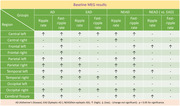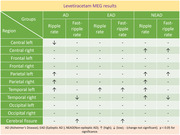# High Frequency Oscillations in Epileptic and Non‐Epileptic Alzheimer’s Disease Patients and the Effect of Levetiracetam on the Oscillations

**DOI:** 10.1002/alz.089593

**Published:** 2025-01-09

**Authors:** Vishnu Shandilya Mungamuru Chenchu, Kwaku Addo‐Osafo, Kamalini G Ranasinghe, Mohamad Shamas, Richard Staba, Srikantan Nagaragan, Keith Vossel

**Affiliations:** ^1^ University of California, Los Angeles, CA USA; ^2^ University of California San Francisco, San Francisco, CA USA; ^3^ University of California, San Francisco, CA USA

## Abstract

**Background:**

Altered network synchronization and rhythmic neural activity is observed in Alzheimer’s disease (AD). Spontaneous epileptiform activity and/or seizures occur in an estimated 60% of AD cases, and having AD increases the likelihood of seizures when compared with people without dementia. Thus, network hyperexcitability can be an early feature and helpful for diagnosis and treatment. However, it is unknown if biomarkers of epileptogenicity are detectible in AD, and if these biomarkers are affected by the anti‐seizure drug levetiracetam (LEV). One important biomarker of epileptogenicity is high‐frequency oscillations (HFOs).

**Method:**

To measure HFOs, we used 10‐min magnetoencephalography (MEG) recordings (275‐channel, sampling rate 1200–2400 Hz) during awake resting periods in participants with AD and healthy controls. Recordings from 14 AD (6 non‐epileptic AD (NEAD, median age: 60.8, 2M/4F) and 8 having subclinical epileptic activity (EAD, median age: 54.9, 5M/3F)) and 8 control (median age: 71, 5M/3F) participants were analyzed using two software scripts: Delphos and a custom‐made script for detection of HFOs. LEV 125 mg twice‐a‐day or placebo was administered for 4 weeks in between two MEG recordings, and 4 weeks of washout before switching LEV/placebo phases for each participant.

**Result:**

HFOs were categorized into ripples (80 to 250 Hz) and fast‐ripples (250 to 500 Hz). At baseline, AD participants, including both NEAD and EAD, had higher ripple rate (RR) and higher fast‐ripple rate (FR) than controls in several regions (p<0.05, paired t‐test/ANOVA‐Tukey). Additionally, compared to EAD, NEAD had higher RR in left‐frontal, left‐temporal, and cerebral fissure regions, and higher FR in left frontal regions (p<0.05, ANOVA‐Tukey). In both EAD and NEAD, LEV increased RR in left‐temporal and bi‐parietal regions, but decreased RR in the left central region (p<0.05, paired t‐test). In both AD groups, LEV increased FR in a few regions, but it decreased FR in right temporal region in NEAD.

**Conclusion:**

AD had a high level of HFOs, with higher numbers observed in non‐ epileptic AD. LEV resulted in region‐specific increases in HFOs in both NEAD and EAD. Thus, HFOs can function as a biomarker of hyperexcitability in AD; however, their contribution to the epileptic phenotype in AD requires further investigation.